# Supplementary material for: MetaQTL: a package of new computational methods for the meta-analysis of QTL mapping experiments
Source: BMC Bioinformatics. 2007 Feb 8;8:49. doi: 10.1186/1471-2105-8-49 (PMC1808479; doi:10.1186/1471-2105-8-49)
Supplement: Additional File 7 — MetaQTL Package : jar file and tutorial. This Zip archive contains both the MetaQTL JAR file and the files of the tutorial. [file 1471-2105-8-49-S7.zip › org.inra.metaqtl/doc/org/metaqtl/bio/class-use/IBioCross.html]

Uses of Interface org.metaqtl.bio.IBioCross


|  |  |  |
| --- | --- | --- |
| **Overview** \| **Package** \| **Class** \| **Use** \| **Tree** \| **Deprecated** \| **Index** \| **Help** | |  |
| PREV   NEXT | **FRAMES**    **NO FRAMES**     **All Classes** |  |


---


## **Uses of Interface org.metaqtl.bio.IBioCross**

| Packages that use IBioCross | |
| --- | --- |
| **org.metaqtl** |  |
| **org.metaqtl.adapter** |  |
| **org.metaqtl.bio.entity** |  |

Uses of IBioCross in org.metaqtl

| Methods in org.metaqtl with parameters of type IBioCross | |
| --- | --- |
| `void` | `ChromCluster.addChromosome(IBioLGroup group, IBioCross crossDesign, int mappingFunction, int mappingUnit, boolean rescale, boolean skeleton)`             Add a chromosome to the cluster |
| `void` | `MapCluster.addChromosome(IBioLGroup group, IBioCross crossDesign, int mappingFunction, int mappingUnit, boolean rescale, boolean skeleton)`             Add a chromosome to the map cluster framework. |

Uses of IBioCross in org.metaqtl.adapter

| Methods in org.metaqtl.adapter with parameters of type IBioCross | |
| --- | --- |
| `static Chromosome` | `ChromosomeAdapter.toChromosome(IBioLGroup group, IBioCross crossDesign, int mappingFunction, int mappingUnit)`             This method converts a linkage group into a chromosome of type `Chromosome` with the given cross design and the given mapping context. |

Uses of IBioCross in org.metaqtl.bio.entity

| Classes in org.metaqtl.bio.entity that implement IBioCross | |
| --- | --- |
| `class` | `CrossPopulation`             Class Description Here |

---


|  |  |  |
| --- | --- | --- |
| **Overview** \| **Package** \| **Class** \| **Use** \| **Tree** \| **Deprecated** \| **Index** \| **Help** | |  |
| PREV   NEXT | **FRAMES**    **NO FRAMES**     **All Classes** |  |


---
